# Acute Pain Therapy in Postanesthesia Care Unit Directed by Skin Conductance: A Randomized Controlled Trial

**DOI:** 10.1371/journal.pone.0041758

**Published:** 2012-07-27

**Authors:** Michael Czaplik, Christa Hübner, Markus Köny, Julia Kaliciak, Fatima Kezze, Steffen Leonhardt, Rolf Rossaint

**Affiliations:** 1 Department of Anesthesiology, University Hospital Aachen, Aachen, Germany; 2 Philips Chair for Medical Information Technology, RWTH University Aachen, Aachen, Germany; The James Cook University Hospital, United Kingdom

## Abstract

**Background:**

After surgery, effective and well-directed acute pain therapy is a necessary and integral part of the overall treatment plan. Generally, the assessment of pain intensity depends on a patient’s self-evaluation using scoring systems such as numeric rating scales (NRS, 0 to 10). Recently, a “Pain Monitor” was commercially provided which is based on measurements of fluctuations of skin conductance (NFSC). In this randomized, controlled, single-blind trial, possible benefits of this certain device were studied.

**Methods:**

Postoperative patients (n = 44) were randomly assigned to a test or a control group during their stay in the postanesthesia care unit (PACU). All patients were treated and monitored according to internal hospital standards. Whereas all patients systematically evaluated their pain each 15 min, test group patients were additionally addressed when NFSC exceeded a predefined level. In cases of NRS≥5 during a routine elevation or in between, pain relief was achieved by standard procedures irrespective of group allocation.

**Results:**

During their stay in PACU, both test and control groups experienced a significant decrease in NRS as a consequence of pain therapy. No significant differences in mean NRS or in NFSC values were found between the test and control groups. No correlation was observed between NRS and NFSC.

**Conclusion:**

Postoperative patients experience diverse stressors, such as anxiety, disorientation, shivering, sickness and pain. Although the application of continuous pain monitoring would be meaningful in this clinical setting, the tested device failed to distinguish pain from other stressors in postoperative adult patients.

**Trial Registration:**

German Clinical Trials Register DRKS00000755.

## Introduction

Despite standard interventions, postoperative pain remains a major inconvenience for approximately 50% of patients [1]. In 10–50% of patients, acute pain becomes chronic [2]. Adequate treatment, which contributes significantly to the postoperative stress reaction, can reduce morbidity and mortality [3], [4]. Hence, an accurate and timely assessment of pain levels is crucial to reduce the affected time period [5], [6]. Because it is a subjective phenomenon, patients perform self-evaluation of pain intensity. Several scoring systems are available for this purpose [7]–[9], all of them depend on the patient’s cooperation, vigilance and cognitive ability. Accurate assessments of pain intensity are difficult, especially for young children. Moreover, self-evaluation is not feasible for unconscious or delirious patients. Therefore, an objective measuring device would be of great value to improve the management of postoperative pain relief [10], [11].

As long ago as 1967, the measurement of skin electrical properties was found to be a promising method to quantify hypnosis. Nisbet et al. stated that changes in skin conductance (SC) were already mentioned in 1888 [12]. Recently, several studies were performed to compare skin conductance with other parameters in intra- and postoperative scenarios concerning the evaluation of vegetative stress. Whereas the amplitude of SC shows wide individual variability, the number of fluctuations in skin conductance over time (NFSC) has been determined to be a sensitive and specific parameter for nociceptive stimuli, particularly in comparison with hemodynamic parameters [13]–[15]. In addition, several studies have confirmed a high correlation between NFSC and self-reported pain intensity on a numeric rating scale (NRS, ranging from 0 =  “no pain” to 10 =  “worst possible pain”). Because of diverse cut-off values, time periods and patient populations, sensitivities and specificities varied for the discrimination of low, moderate and severe pain [16], [17]. Nevertheless, multiple factors influence the skin conductance of postoperative patients, such as disorientation, shivering, anxiety, sickness and interactions with other patients and staff. Until now, there is no evidence that acute pain therapy controlled by SC in the postanesthesia care unit (PACU) can improve patient outcomes.

The aim of our study was to evaluate the possible benefits of continuously monitoring pain intensity for postoperative patients by measuring NFSC with a commercially provided device. The primary outcome parameter of this randomized, controlled, single-blind trial was the mean pain intensity of postoperative patients during their stay in PACU as measured by NRS.

## Methods

### 1. Study Population, Ethics and Groups

This randomized, controlled, single-blind trial took place in PACU at the University Hospital Aachen after approval by the local ethics committee (EK 063/11) and registration at the German Clinical Trials Register (DRKS00000755). From April, 28th to June, 10th, 2011, 44 patients were informed preoperatively about the study via a standardized leaflet and provided written consent for their participation. The protocol for this trial and supporting CONSORT checklist are available as supporting information; see Checklist S1 and Protocol S1. The inclusion criteria were age above 18 years, ability to give consent, surgical intervention scheduled for at least 90 min, general anesthesia and an intended postoperative stay in PACU. By contrast, intended stay at the intensive care unit (ICU), implanted pace makers or cardioverter-defibrillators, therapy with catecholamines, regional anesthesia and patient-controlled analgesia were the exclusion criteria. Immediately after surgery patients were assigned to either the “supported by technique” (TC) or the control group (CO) using a randomization list. Randomization was carried out with Randlist V1.2 (Datinf GmbH, Tübingen, Germany) and generated by an independent colleague. While the medical attendants’ behavior differed between CO and TC group, patients of both groups were monitored equally in order to stay blinded.

### 2. Measurement, Data Acquisition and Study Protocol

**Figure 1:**
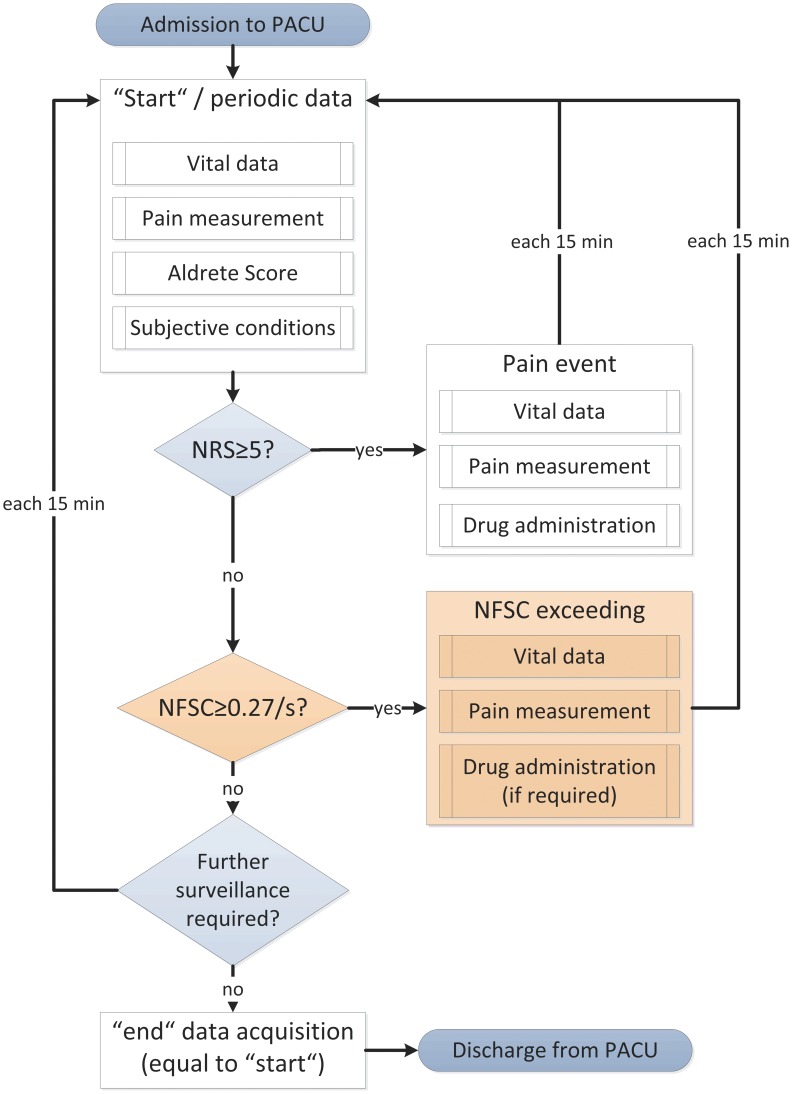
Flow chart of study protocol. Data acquisition was performed after admission to postanesthesia care unit (PACU), furthermore each 15 min, in cases of pain pronouncement and prior to discharge from PACU. Only in TC group, NFSC exceeding was regarded and pain relief established if required (shapes shaded in light-red).

**Figure 2:**
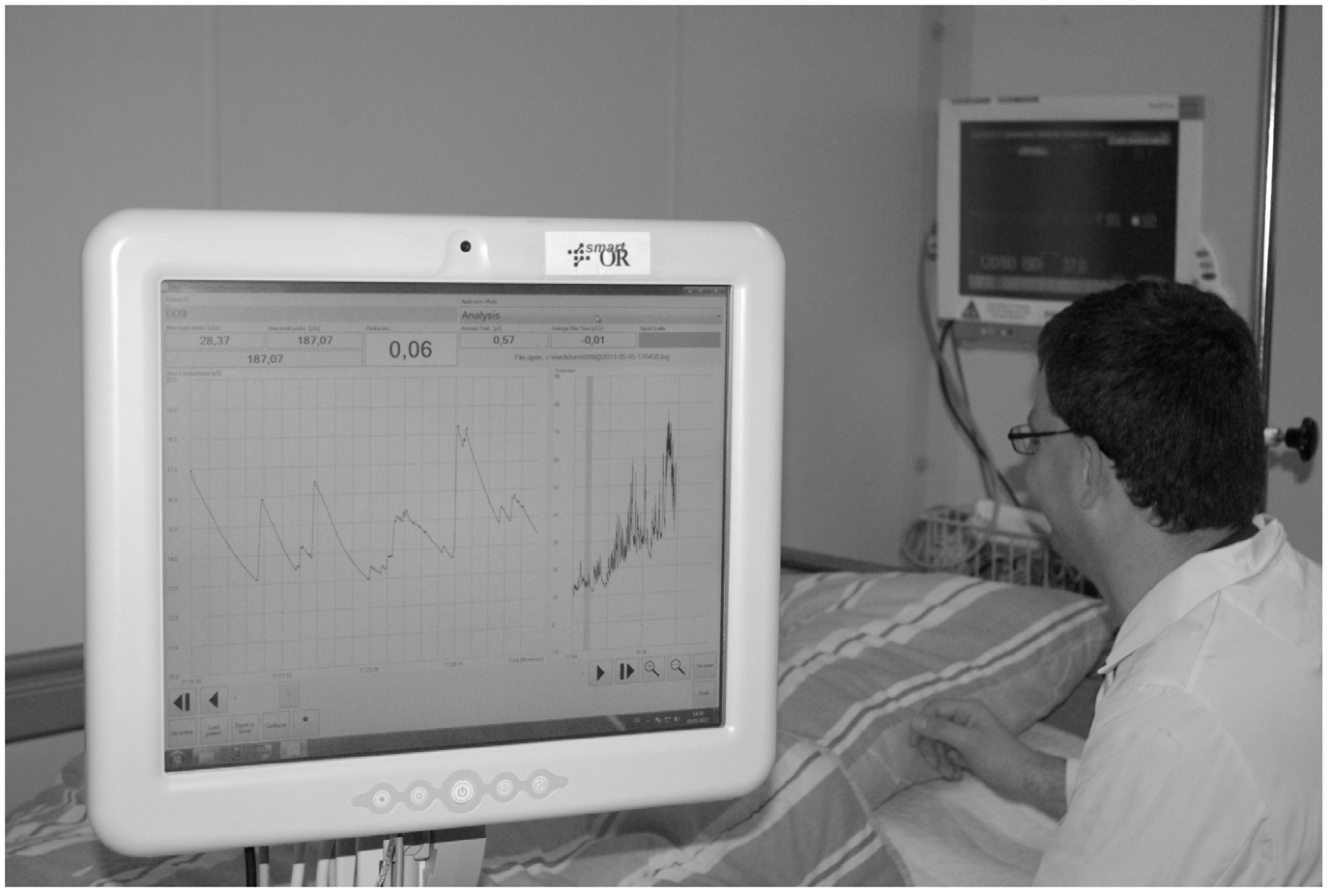
Application of the pain monitor at postanesthesia care unit. For the clinical trial the software mode “post-operative and intensive care” was used. For TC group acute pain therapy was supported by “peaks/sec” value which is calculated in real-time.

After admission to PACU, a standardized data sheet titled “beginning of treatment” was used. In this form, vital data, initial NRS (0–10) and NFSC score, modified Aldrete score (0–10 points: sum of motoric activity, breathing, blood pressure, consciousness, oxygen saturation; 0–2 points each) [18] and ratings of subjective conditions (table 1) was documented. Subsequently, these data sets were collected every 15 min using another tabular sheet titled “periodic data collection” for the duration of the stay in PACU (figure 1). For this purpose, patients were connected to a Philips IntelliVue MP30 monitor (Philips Electronics N.V., Amsterdam, Netherlands) for continuous vital data measurement (ECG, noninvasive blood pressure every 15 min, oxygen saturation) and to the “Med-Storm Pain Monitor” (Med-Storm Innovation AS, Oslo, Norway). The provided software “Skin Conductance Measurement System 1.0′′ was used for real-time calculation of NFSC in application mode “post-operative and intensive care” (figure 2). In addition to the manually derived NFSC values, for comparison reasons mean values (with time frames of 30 s and 60 s at the defined time points) were calculated afterwards from the captured SC data by the same program.

**Table 1:** Subjective conditions, assessed by questioning/observation.

Item	Value
Vigilance	0: awake, 1: tired, 2: sleeping
Well-being	0: poor, 1: fair, 2: good, 3: excellent
Energy level	0: normal, 1: fair, 2: poor
Agitation	0: calm, 1: uneasy, 2: agitated
Nausea/Vomiting	0: no, 1: nausea, 2: vomiting

In cases of pronounced pain (NRS≥5) detected during a routine evaluation or in between, pain relief was achieved by standard procedures regardless of group allocation. Generally, in addition to a non-opioid analgesic 0.05 to 0.1 mg per kg body weight of piritramide was administered and titrated to effect. These events were recorded on an additional sheet titled “pain aggravation” to document vital data, NRS score, NFSC value and the antinociceptive effect after 5 min.

When NFSC reached the predefined threshold of 0.27/s for at least 30 s, which corresponds to NRS≥4 or “other stressors” as detailed in the Med-Storm Pain Monitor manual, patients in the TC group were asked whether there was an initiating pain, which was treated when identified (table 2). These events and the patients’ self-evaluations were documented on a sheet titled “NFSC exceeding”, that was analogous to the “pain aggravation” sheet. For the CO group, NFSC values were ignored.

**Table 2:** Allocation of NFSC to pain intensity or other reasons.

Color coding	NFSC limit	Reason/suggestion
White	0.00 to 0.07 peaks/s	No pain
Light yellow	0.13 to 0.21 peaks/s	No pain or VAS 1–3
Yellow	0.27 peaks/s	Patient is active, can be pain VAS 4–5 or other stressors
Orange	0.33 peaks/s	Patient is possibly in pain, VAS 6–8, go and evaluate the situation
Red	0.40 to 0.70 peaks/s	The patient is probably in pain, VAS 8–10, go and find out how to help the patient

Prior to discharge from PACU, a final data acquisition titled “end of treatment” was performed in a manner similar to the first evaluation (figure 1).

### 3. Endpoints

The primary endpoint was defined as the mean NRS score expressed by the patient during the whole stay in PACU. Secondary endpoints were the ratings of subjective conditions (vigilance, well-being, energy level, agitation and nausea), the duration of stay in PACU, total dose of administered analgesics and mean Aldrete score.

### 4. Statistical Methods

The number of required subjects was determined by a prior power analysis using nQuery Advisor 7 (Statistical Solutions, Saugus, MA, USA): A priori, two-tailed t test, power 0.8, α = 0.05. The hypothesis was an expected reduction of NRS mean value by 20% and a standard deviation of ±25% in each group.

SPSS Statistics 19 for Windows (SPSS Inc., IBM Business Analytics Software, Armonk, NY, USA) was used for statistical analysis. Apart from Kolmogorov-Smirnov test and Spearman’s correlation, Mann-Whitney-U-Test was used for independent data samples (TC versus CO group) and Wilcoxon Test for repetitive parameter assessments (PACU arrival, discharge and in between). Mean plus/minus standard deviation is given for all normally distributed parameters, otherwise median and interquartile range is stated. MedCalc (MedCalc Software BVBA, Mariakerke, Belgium) was applied for ROC analysis that was performed in addition to provided endpoints.

## Results

**Figure 3:**
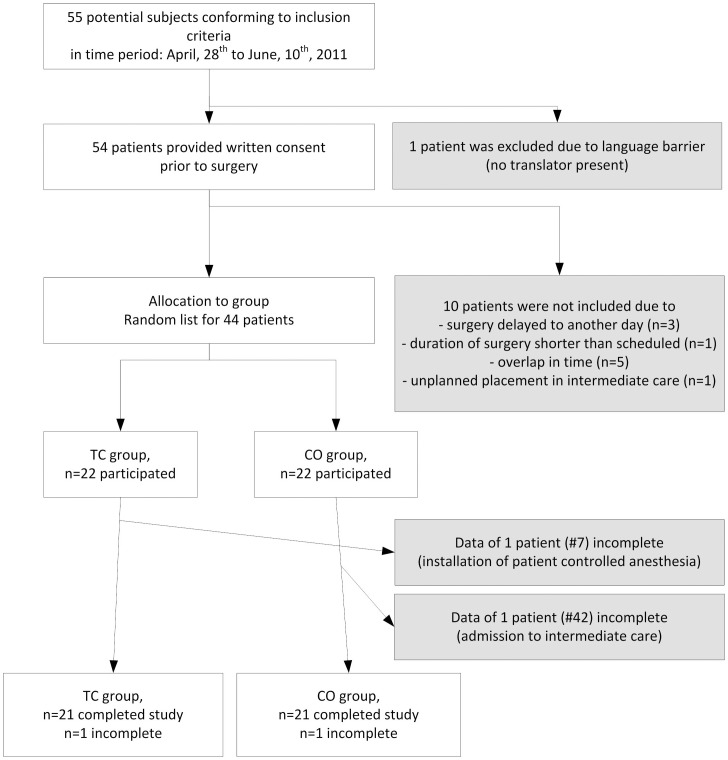
Flow chart.

In total, 55 patients were screened as possible subjects by studying the surgery schedule for the next day. After arrival at the PACU, 44 of them were evenly allocated to the CO and TC group (figure 3).

After group allocation, the study groups showed similar characteristics with respect to sex (14 females (TC) vs. 16 females (CO)), age (54.6±14.4 (TC) vs. 59.1±14.4 (CO)), number of total intravenous anesthesia (8 cases each) and vital data (table 3).

**Table 3:** Vital data and scores in postanesthesia care unit (PACU).

	Arrival at PACU			Mean values during stay			Discharge from PACU		
	TC group	CO group	P	TC group	CO group	P	TC group	CO group	p
NRS score	5 [2]–[6]	4 [2]–[5]	0.53	2.8 [1.4–4.2]	3.1 [1.9–4.3]	0.13	3 [2]–[4]	2 [0–2]	**0.03**
Blood pressure systolic (mmHg)	135 [113–166]	144 [125–156]	0.43	132 [118–157]	139 [121–152]	0.62	135 [118–159]	138 [118–151]	0.88
Blood pressure diastolic (mmHg)	76 [65–85]	78 [65–87]	0.82	72 [59–81]	74 [65–85]	0.32	72 [67–83]	71 [64–81]	0.87
Heart rate (/min)	78 [69–86]	80 [71–90]	0.47	78 [68–88]	77 [70–85]	0.57	75 [67–85]	74 [70–88]	0.74
NFSC (/min)	0.07 [0–0.07]	0 [0–0.07]	0.59	0.07 [0.02–0.12]	0.10 [0.05–0.16]	0.30	0.10 [0.02–0.18]	0.13 [0.13–0.20]	0.41
Aldrete score	9 [7]–[10]	8.5 [7.3–9.8]	0.73	9 [8]–[10]	9 [7.25–10]	0.24	10 [8]–[10]	9 [9]–[10]	0.25
Vigilance	1 [0–2]	1 [1]–[2]	0.45	1 [0–2]	1 [1]–[2]	0.26	0 [0–1]	1 [0–1]	0.98
Well-being	1 [0.3–1]	1 [0–1]	0.80	1 [1–1.8]	1 [1–1]	0.30	1 [1]–[2]	1 [1–1]	0.20
Energy level	1 [1–1.8]	1 [1]–[2]	0.44	1 [1–1]	1 [1]–[2]	0.26	1 [1–1]	1 [1–1]	0.81
Agitation	0 [0–0]	0 [0–0]	n.a.	0 [0–0]	0 [0–0]	0.96	0 [0–0]	0 [0–0]	0.31
Nausea	0 [0–0]	0 [0–0]	0.32	0 [0–0]	0 [0–0]	**0.02**	0 [0–0]	0 [0–0]	0.33

Pain intensity (NRS score), vital data, number of skin fluctuations (NFSC) and clinical scores are given for three points. Data are stated as median values and interquartile ranges,

During the stay in PACU, no significant differences were observed between TC and CO groups in either primary or secondary endpoints. Furthermore, the hypothesized difference of 20% concerning the primary endpoint was not achieved. In particular, mean NRS score was 2.8 (IQR 1.4–4.2) and 3.1 (IQR 1.9–4.3; p = 0.13) respectively- a relative difference of 9.6%. Additionally, NFSC did not differ at any time. After discharge from PACU, NRS scores differed significantly between TC and CO groups (table 3).

As expected, pain intensity decreased significantly during the stay in PACU in both TC (5 (IQR 2–6) vs. 3 (IQR 2–4), p<0.05) and CO group (4 (IQR 2–5) vs. 2 (IQR 0–2), p<0.01) showing no inter-group differences. Concerning subjective condition scores, vigilance differed in the CO group (p = 0.04) and well-being differed in the TC group (p = 0.02) between arrival at PACU and discharge. Administered analgesics were similar in both groups. Total number of severe pain episodes requiring piritramide administration was 33 in CO versus 35 in TC group (p = 0.75). Total dose of piritramide was higher by tendency in TC group (table 4).

**Table 4:** Postoperative pain relief.

	TC group	CO group	P
Stay at postanesthesia care unit (min)	78±36	96±52	0.25
Drug administration (total dosage):			
▪ piritramid (mg)	273.5	235.0	0.71
▪ parecoxib (mg)	200	200	1.00
▪ diclofenac (mg)	200	0	0.15
▪ metamizole (g)	16	24	0.19
▪ paracetamol (g)	2	1	0.55
Number of treatments	35	33	0.75

**Figure 4:**
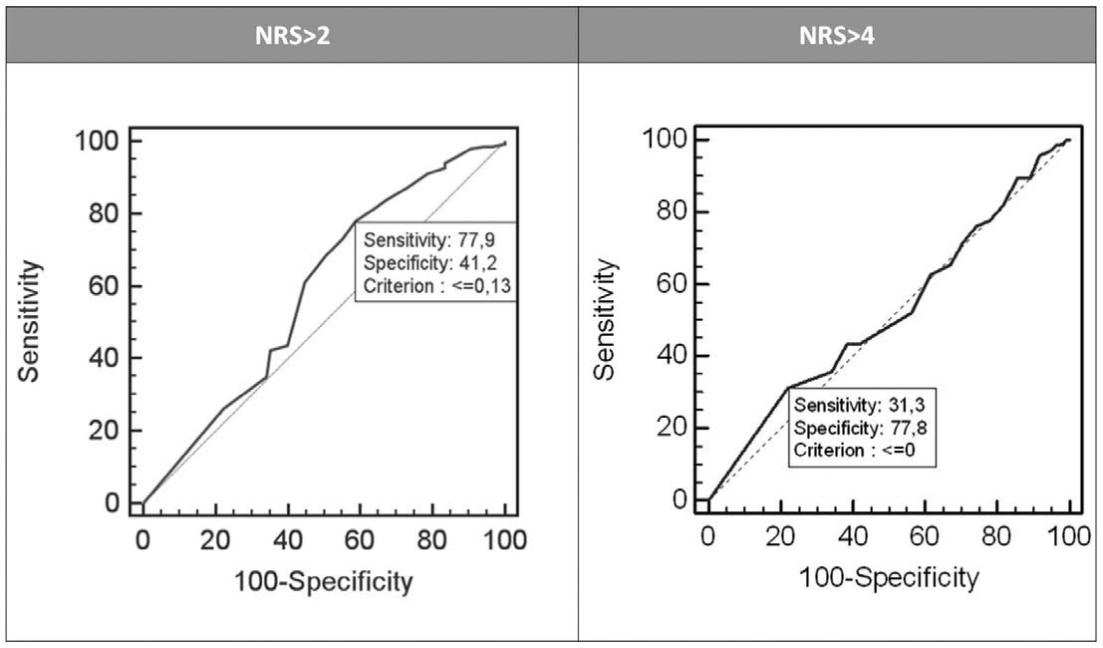
ROC analysis for pain detection with NFSC. Best sensitivity (77.9%) but relatively poor specificity (41.2%) was obtained for the detection of NRS>2 by criterion “number of fluctuations of skin conductance (NFSC) >0.13′′. For higher pain intensities, like NRS>4, sensitivity and specificity were even worse.

No correlations were observed between NRS and NFSC. Manually derived NFSC and mean NFSC values (time period of 60 s) correlated according to Pearson’s method (r = 0.571, p<0.001). Regarding ROC analysis, best sensitivity (77.9%) and specificity (41.2%) were obtained for the detection of NRS>2 with the criterion NFSC>0.13/min (figure 4).

## Discussion

In this randomized controlled trial, we examined whether the measurement of NFSC is an effective method to improve acute pain therapy in postoperative patients. As our primary endpoint, mean NRS scores during patients’ recovery phase was observed and compared. No differences between the TC and CO groups were observed for this parameter or any secondary endpoints. In particular, the hypothesized decrease of 20% regarding the mean NRS score in TC group was not achieved.

While NFSC did not differ between the time points within the groups, the NRS score was significantly higher in the TC group at the end of PACU care. In both groups, pain intensity decreased significantly during the stay in PACU to a tolerable value of lower than three as expected. All patients had already received analgesics (mostly metamizole, NSAID and piritramide (0.05–0.10 mg/kg body weight)) under anesthesia according to clinical standards (table 4). Therefore, the amount of administered analgesics in PACU was relatively low. Since the administered dose of piritramide in TC group was already higher before group allocation, this finding reflects a higher occurrence of painful surgeries in this group rather than a negative effect of the applied pain monitor.

Concerning subjective condition scores between arrival at PACU and discharge, vigilance increased significantly (p = 0.04) in the CO group and well-being increased in the TC group (p = 0.02). However, assessing all “subjective conditions” items was especially challenging for sleepy or somnolent patients. For sleeping patients, scores were rated by the observations of the medical attendant.

Although the software used for online NFSC calculation was clearly arranged, user friendly and NFSC values were refreshed each second, these values fluctuated frequently and were therefore difficult to determine. To overcome this difficulty, the attendant was instructed to observe the NFSC for at least 30 s to identify and document the predominant value. This approach was intricate and error-prone. Hence, manually derived values were *post-hoc* compared with mean NFSC values over time frames of 15 s, 30 s and 60 s (NFSC60). The strongest correlation found was between manual NFSC and calculated NFSC60.

Nevertheless, no significant correlation was observed between NRS and NFSC. Consequently, the sensitivity and specificity for pain detection obtained via ROC analysis were weak. Thus, the area under the curve (AUC) is barely better than 0.5 which correspond to flipping a coin.

There are several possible explanations for these findings. Changes in NFSC were already caused by addressing the patient to assess pain intensity or subjective sensations. Although NFSC was documented prior to questioning, this fact implies a strong interaction of skin conductance (or its measurement) with other factors. Even artifacts such as moving the arm or the hand to which the electrodes were attached led to errors in measurement. Moreover, not only pain but also stress in general (due to more or less sedation, alertness, noise, anxiety, trouble, disorientation, etc.) affects patients after surgery. Unfortunately, these factors are manifold and almost unpredictable.

Recently, various clinical trials of the ability of SC measurement to detect stress came to different conclusions [11]. Generally, the specific scenarios (operation room, intensive care unit, PACU and laboratory) and the employed study population (anesthetized patients, sedated patients, children, postoperative awake patients and healthy test persons) have to be considered. Furthermore, the applied stimuli (noxious stimuli, tracheal intubation, endotracheal suctioning) and parameters used for comparison reasons (Bispectral index (BIS), locally developed clinical indices, hemodynamic parameters) have to be considered. Besides there are other factors that affect the validity of SC parameters, such as administration of neostigmine to reverse neuromuscular blockade [19].

In 1981, Wallin described a correlation between neurophysiological reactions and variations in electrodermal activity [20]. Since that time, several authors have concluded that skin conductance peaks occur approximately 1–2 s after stimulation, depending on the sympathetic nerve activity and therefore, it can be used for the assessment of pain and nociception [14]. Additionally, SC measurement is frequently used for the estimation of sedation. The fewer (influencing) affecting factors, the more successful and accurate is the assignment of a stimulus (such as an initiating pain level) to a SC variation. Thus, in our point of view, this technique should work best in sedated patients. Moreover, an application in sedated, unconscious or uncooperative patients should be for sure the primary purpose. A more titrative drug use could lead to a reduction of opioids, less adverse events and finally to a fastened transfer to the ward. Incidentally, measurement of surgical stress index (a technique that is based on plethysmography) with Carescape Modular Monitor (GE Healthcare, Chalfont St Giles, United Kingdom) is intended for use in unconscious and fully anesthetized adult (>18 years old) patients during general anesthesia [21].

Several intraoperative trials have indicated that palmar SC reaches a high sensitivity and specificity (up to 86% each) in the detection of noxious and awakening stimuli relative to hemodynamic parameters and BIS [13], [22]–[26]. However, a smaller number of intraoperative studies did not confirm these results and have even suggested the inferiority of SC as compared with BIS [27] or hemodynamic parameters and plasma noradrenaline levels [28]. Similarly to intraoperative probands, sedated ICU patients are isolated from various factors. NFSC was proposed as an objective supplement to the COMFORT sedation score and was superior to heart rate and blood pressure in artificially ventilated children [29]. In a similar finding, NFSC successfully mirrored the stress response from heel sticks in preterm infants [30].

In contrast to these results, trials that took place in PACU show a more heterogeneous pattern. Similar to our results, NFSC failed to reflect acute postoperative pain in a trial from Ledowski et al. in 2009 [10] and Choo et al. in 2010 [31], although other studies came to more favorable conclusions with high sensitivities for pain detection [15], [17], [32]. However, one of these studies concluded that NFSC may be influenced by factors other than pain, so that it should not be used as a sole tool for pain assessment [15].

Several limitations of this study must be addressed. Whereas we used few inclusion criteria, a more homogeneous study population with regard to psychiatric diseases, chronic pain and more similar surgeries could reveal more subtle differences between study groups. Patients in the CO and TC group were interrogated every 15 min and were treated with analgesics if their pain score was moderate. Certainly, on the one hand this design makes a differentiation difficult but on the other hand, this is a very common procedure in PACU that we did not want to downgrade for the study. Besides, we didn’t use cognitive tests or questionnaires. Concerning this matter, we didn’t expect relevant differences and didn’t want to influence normal PACU processes too deeply. Patients were blinded according to the assigned group; the medical attendant who documented the data periodically was not. However, in the CO group, the NFSC value was only scored every 15 min (but not in between) for documentation, thereby minimizing any impact on the attendant’s behavior.

An objective technique to assess the pain intensity of communication-hindered patients remains a promising but elusive goal. Although the application of continuous pain monitoring would be meaningful especially in postoperative patients, the tested device failed to distinguish pain from other stressors.

## Supporting Information

Protocol S1 **Original study protocol prepared for clinical trial translated from German.**
(DOCX)

Checklist S1 **Checklist filled out according to CONSORT criteria.**
(DOC)
