# Supplementary material for: Acute Pain Therapy in Postanesthesia Care Unit Directed by Skin Conductance: A Randomized Controlled Trial
Source: PLoS One. 2012 Jul 27;7(7):e41758. doi: 10.1371/journal.pone.0041758 (PMC3407175; doi:10.1371/journal.pone.0041758)
Supplement: Protocol S1 — Original study protocol prepared for clinical trial translated from German. (DOCX) [file pone.0041758.s001.docx]

|  |
| --- |

| **Study protocol** |
| --- |
| Support of acute pain therapy through continuous algesia measurement of post-operative patients in the anesthesia recovery unit (ARU) |
|  |
| Post-operative pain affects recovery, tends to chronification and is an extremely unpleasant phenomenon for the patient. Therefore, palliative care for acute pain assumes special importance especially in ARE. However, characterisation of this subjective experience is difficult. Until recently, one can only "measure" the pain based on the indication of the pain intensity on a "Numeric Rating Scale" (NRS) by the patient. Based on skin conductance, the "Number of Fluctuations of Skin Conductance per Second" (NFSC) can be determined. This shows high correlation with the value on the NRS and can be raised irrespective of the cooperation of the patient. In addition, the "Surgical Stress Index" (SSI) was examined previously in studies on its capability to quantify perioperative stress.  The goal of this randomised, controlled single-blind study is the investigation of the suitability of NFSC and SSI for supporting palliative care in ARU. The primary endpoint to be examined represents the average NRS value specified, further endpoints are the retention time in ARU, the Aldrete score, the satisfaction of the patient, the entire quantity of analgesics administered as well as the course of the salivary cortisol level. |
|  |
|  |
| **2011/03/22** |
|  |

**Study protocol**

**General Information**

## Study title

Support of acute pain therapy through continuous algesia measurement of post-operative patients in the anesthesia recovery unit (ARU)

## Name und Adresse des Sponsors

University Hospital of Aachen, Clinic for Anaesthesiology, Pauwelsstr. 30, 52074 Aachen, Germany

## Authorised signatories

Dr Michael Czaplik

Prof Dr Rolf Rossaint

## Principal investigators

Dr Michael Czaplik

Fatima Kezze

Dr Julia Kaliciak

Prof Dr Rolf Rossaint

## Doctoral candidate

Christa Hübner

## Other clinics and institutes involved

None

## Summary

Post-operative pain affects recovery, tends to chronification and is an extremely unpleasant phenomenon for the patient. Therefore, acute pain therapy assumes special importance especially in ARR. However, characterisation of this subjective experience is difficult. Until recently, one can only "measure" the pain based on the indication of the pain intensity on a "Numeric Rating Scale" (NRS) by the patient. Based on skin conductance, the "Number of Fluctuations of Skin Conductance per Second" (NFSC) can be determined. This shows high correlation with the value on the NRS and can be raised irrespective of the cooperation of the patient. In addition, the "Surgical Stress Index" (SSI) was examined previously in studies on its capability to quantify perioperative stress.

The goal of this randomised, controlled single-blind study is the investigation of the suitability of NFSC and SSI for supporting acute pain therapy in ARU. The primary endpoint to be examined represents the average NRS value specified, further endpoints are the retention time in ARU, the Aldrete score, the satisfaction of the patient, the entire quantity of analgesics administered as well as the course of the salivary cortisol level.

# Background information

## Overview over know studies

The "International Association for the Study of Pain" defines pain as an unpleasant sensory and emotional experience that is associated with an actual or potential tissue damage. Moreover, it is also regarded as an emotional experience because it is always unpleasant [^[[1]](#endnote-2)^].

Post-operative pain occurs in approximately 50% of the patients [^[[2]](#endnote-3)^]. Acute post-operative pain becomes chronic in 10-50% of the cases and can become serious in 2-10% of these patients [^[[3]](#endnote-4)^]. Furthermore, pain can suppress immune response and can cause unfavourable consequences; increased incidence of metastasis could also be found [^[[4]](#endnote-5)^]. The treatment of pain can reduce the morbidity and mortality, as pain contributes considerably to post-operative stress response [^[[5]](#endnote-6)^, ^[[6]](#endnote-7)^]. The timely detection of post-operative pain is decisive for shortening the time until pain investigation [^[[7]](#endnote-8)^, ^[[8]](#endnote-9)^].

Because pain is always subjective, the evaluation of the pain intensity must be based on self-assessment of the patient. Several scoring systems are available for this purpose [^[[9]](#endnote-10)^, ^[[10]](#endnote-11)^, ^[[11]](#endnote-12)^].

However, all scoring systems that depend on cooperation of the patient have obvious limitations in patients with speech / hearing disorders or language-related problems as well as patients with cognitive disorder. The exact detection of the pain intensity in small children using this method is impeded and fails in unconscious or delirious patients. An objective measuring instrument that illustrates the pain or the the associated stress would thus be of great benefit to improve management of post-operative pain [^[[12]](#endnote-13)^].

As early as 1967, the measurement of changes to electric characteristics of skin in combination with other physiological parameters was described as a prospective method to be able to determine the degree of sedation of a patient. In a study by Nisbet et al., it was mentioned that the change to the skin resistance was first documented as early as 1888 and the changes to the skin potential in 1890 [^[[13]](#endnote-14)^]. Therefore, these parameters were investigated obviously for more than a century.

In the recent years, various studies were carried out to examine the Skin Conductance (SC) in comparison with other parameters in the intra and post-operative setting for measurement of vegetative stress.

While the amplitude of SC is subject of strong inter-individual differences, the occurred Number of Fluctuations of Skin Conductance per Second (NFSC) was increasingly seen as a parameter that is sensitive and specific to nociceptive stimuli. In the process, NFSC responds especially more sensitively and specifically than a haemodynamic parameter (heart rate and blood pressure) [^[[14]](#endnote-15)^, ^[[15]](#endnote-16)^].

However, a positive correlation could be shown between NFSC and the epinephrin level, as well as blood pressure. It could also be partly shown that NFSC responds faster to the awakening of a patient than the bispectral index (BIS) [^[[16]](#endnote-17)^, ^[[17]](#endnote-18)^, ^[[18]](#endnote-19)^].

For distinction between different scenarios in the waking-up process, Lederowski et al. found similar changes in NFSC and BIS that were clearly superior to the haemodynamic parameters [^[[19]](#endnote-20)^].

In addition, they were able to show a high correlation between NFSC and the value on "Numeric Rating Scale" (NRS) that subjectively reflects the pain perceived by the patient. Regarding the distinction between mild, medium and strong pain, a high sensitivity and acceptable specificity was reached in several studies [^[[20]](#endnote-21)^, ^[[21]](#endnote-22)^]. Besides this achievement, another team was also able to determine a cut-off value for NSFC, which determines - with a sensitivity of 90% and a specificity of 64% - the time points, in which the patient perceived moderate and/or strong pains [^[[22]](#endnote-23)^].

Gjerstad et al. observed that the NSFC allows better prognosis of clinical stress during intubation than the "Response Entropy" (RE) - a parameter of the Electromyography (EMG). In contrast to RE, NSFC responded also to tetanic stimulation. This response was weakened when opioids were administered in addition to propofol [^[[23]](#endnote-24)^].

The "Surgical Stress Index" (SSI) was developed by Huiku et al. in 2006. The goal was to develop a simple clinical index that includes only the most plausible physiological variables for operative stress to enable an appropriate algesia measurement.

In their first study, they found that the average SSI level was high, if the remifentanil dosage, related to the stimulus, was inadequately low and vice versa.

Ideally, the index indicated both slow changes to the level of anti-nociceptive drugs in blood or the intensity of the surgical stimulation as well as sudden acute stimuli [^[[24]](#endnote-25)^, ^[[25]](#endnote-26)^].

Furthermore, it was found in a study that SSI allows better measurement of the autonomous response to a tetanic tissue-damaging stimulus during specific hypnotic and analgetic conditions then the "State Entropy" (SE, parameter from EMG), the RE, heart rate (HR) or the "pulse wave amplitude" from pulse oximetry (PPGA) [^[[26]](#endnote-27)^].

Kallio et al. compared SSI and PPGA with other stress parameters and concluded that both variables are sensitive indicators of operative stress in children. The endotracheal intubation caused changes to all stress-related variables recorded: SSI, PPGA, HR, non-invasive blood pressure (NIBP, RE and SE). The surgical intervention was accompanied by a significant increase in SSI and reduction in PPGA [^[[27]](#endnote-28)^].

Another established method for evaluation of stress is the measurement of the cortisol level in the saliva. The glucocorticoid level is subject not only to daytime and intra-individual variations, but shows additional variations in the case of acute stress conditions [^[[28]](#endnote-29)^, ^[[29]](#endnote-30)^, ^[[30]](#endnote-31)^]

## Study population

Post-operative patients at the ARU who met the inclusion and exclusion criteria.

# Objective and purpose of the clinical study

The goal of our study is the evaluation of NFSC for continuous monitoring of algesia in post-operative patients at the ARU. Exceeding predetermined threshold values should contribute to the sensitisation of the ARU personnel, who consequently question the patient about pain.

As a consequence, pain is supposed to be treated at an early stage, even before the patient spontaneously indicates pain, as this often happens when very strong pain is experienced (e.g. if NRS > 6). The analgesics dose to be administered may be reduced and the average pain stress (measured as NRS average) may be reduced for the patients, where appropriate.

Due to the higher constancy of an appropriate drug activation level, the entire quantity of administered analgesics and thus, the side-effects are also possibly lower. In addition, the data of the usual pulse oximetry are recorded to calculate the SSI post hoc and to compare with NFSC and NRS. As secondary target parameters, other parameters, such as the cortisol level in the saliva should be recorded for objective detection of stress and the questionnaire to document subjective perception of the patient and evaluated comparing both groups.

# Study design

Randomised controlled single-blind study

Study group: technically supported palliative care (TC)

Control group: conventional palliative care according to standard (CO)

## Primary outcome parameter and secondary parameters

Primary:

- NRS∅ (average NRS score indicated by the patient)

Secondary:

- Satisfaction of the patient (questionnaire)
- Duration of stay at the ARU
- Total dose of analgesics administered
- Incidence of side-effects
- Aldrete score
- Cortisol level in the saliva

4.2 Study design and sequence

Suitable patients are identified using the surgery schedule for the next working day and screened based on inclusion and exclusion criteria.

Following patient information and written consent, the patient is accepted in the study. The randomisation in one of the groups (CO or TC) is done directly after admission of the patient to the ARU following surgical intervention. Refer to the plan for further sequence:

4.3 Sequence and duration of the study phases

Screening and patient information take place on the day before the scheduled operation (approx. 30-60 min. per patient). The stay at the ARU and thus, duration of the record of the study parameters (see plan) is dependent (45-180 min.) only on the post-operative recovery of the patient.

4.4 Proceedings for minimisation of bias

Randomisation, blinding with regard to the patient and ARU personnel, no influence of the in-house ARU standards, especially not the palliative care.

4.5 Discontinuance criteria

- Malfunction of the measuring or recording equipment
- Withdrawal of consent by the patient

4.6 Overview of the study arms

Randomisation in one of the two groups takes place:

Gruppe CO

- Conventional ARU care. This includes measurement and documentation of the vital parameters (blood pressure, respiratory rate, oxygen saturation, pulse) of the patient every 15 minutes. Moreover, the patient is connected to continuous ECG and oxygen saturation measurement.
- Documentation of the vital parameters and the NRS scores is done every 15 minutes.
- When the patient indicates pain, the pain therapy is done according to standard and the vital parameters and the NRS scores documented
- NFSC is measured and recorded, but exceeding the threshold value is not indicated.

Gruppe TC

- Conventional ARU care plus NFSC monitoring
- Documentation of the vital parameters and the NRS scores is done every 15 minutes.
- When the patient indicates pain, the pain therapy is done according to standard and the vital parameters and the NRS scores documented
- NFSC is measured and recorded, exceeding threshold value is visually indicated and also leads to the elevation of the NRS scores and documentation of the vital parameters

# Selection and exclusion of study participants

## Inclusion criteria

- At least 18 years, capable of giving consent
- Surgical intervention, planned minimum duration 90 min, surgical classification at least III
- General anaesthesia
- Planned post-operative stay at the ARU

## Exclusion criteria

- Beta blocker
- Pacemaker / ICD
- Running catecholamine therapy
- Peridural catheter (PDC)
- PCA pump (Patient-Controlled Analgesia)

# Devices and methods used

6.1 Patient monitor

In the ARU, the patient monitor Philips IntelliVue MP30 is used. The parameters required for documentation of the vital parameters and determination of the Aldrete scores are taken from usual monitoring.

6.2 Skin conductance

The skin conductance is measured on the hand using the Medstorm "Stress Detector" (Barberpole, Blomberg, Germany; CE certificate is present) over three adhesive electrodes and is indicated on the display of a medical panel PC.

6.3 Numeric Rating Scale (NRS)

The NRS is the currently most favoured standard for evaluation of pain in awake, cooperative patients [45]. In a study, in which the NRS was compared with the "Facies Pain Scale" and the "Visual Analogue Scale", the patients selected NRS as the preferred method to classify their pain intensity [^[[31]](#endnote-32)^].

The NRS is a 11-element scale, on which 0 indicates "no pain" and 10 "maximum pain". The corresponding value is determined through patient inquiry.

Different formats of the NRS, especially with different number of elements, normally showed high correlations of r² > 0.99 [^[[32]](#endnote-33)^]. The validity and reliability of the NRS in older patients to measure pain with acute or chronic origin was demonstrated [^[[33]](#endnote-34)^, ^[[34]](#endnote-35)^, ^[[35]](#endnote-36)^, ^[[36]](#endnote-37)^].

6.4 Number of Fluctuations of Skin Conductance per Second (NFSC)

Emotional sweating is activated through sympathetic skin nerves and transmitted via muskarinergic acetylcholine receptors; it results largely independent of the ambient temperature as far as it lies within the normal range [^[[37]](#endnote-38)^, ^[[38]](#endnote-39)^]. The skin resistance reduces and thus the skin conductivity (SC) increases until the sweat is reabsorbed and SC drops again [^[[39]](#endnote-40)^, ^[[40]](#endnote-41)^, ^[[41]](#endnote-42)^]. This results in an SC peak, whose magnitude depends on the sympathetic activity [^[[42]](#endnote-43)^33]. It is specific to the triggering stimulus and occurs about every 1-2 s. Its high sensitivity is especially based on the fact that the influence of blood circulation-dependent variations or neuromuscular blockades is only very low [^[[43]](#endnote-44)^, ^[[44]](#endnote-45)^, ^[[45]](#endnote-46)^].

It led to the finding that NFSC is most suitable for the measurement of algesia in comparison with other SC parameters (e.g. average of the absolute SC, amplitude of the SC peaks or their area enclosed by the curve) [^[[46]](#endnote-47)^, ^[[47]](#endnote-48)^].

6.5 Surgical Stress Index (SSI)

Another method for objective measurement of pain is offered by SSI. The index combines evaluation of Heart Rate Variability (HRV) with the analysis of peripheral photoplethysmography (PPG). To this end, a commercially available pulse oximeter (Masimo Radical 7 and Nelcor N600) with analogue signal output for data recording using AD converter is used.

SSI represents a combination of cardiac and peripheral sympathetic tonus [10]. because the amplitude of the PPG, the PPGA depends especially on the sympathetically controlled peripheral vasotonus. In a specified time interval (10 s), the mean Heart Beat Interval (HBI) and PPGA are calculated and standardised post hoc to all recorded values of the respective patient using a legend reference database (many previouslt recorded patients).

Thus, an absolute value for simplification and comparability of different patients was defined for the SSI as:

SSI = 100 – (0.7 x PPGAstandard + 0.3 x HBIstandard) [24].

What is problematic is that exact calculation of the SSI in this manner is possible only retroactively. Intermittent calculations of the SSI solely represent approximations, which become all the more accurate, the longer the previously recorded time span and the recorded stress intensities and/or pain intensities are (more or less in the sense of a multiple point calibration).

6.6 Aldrete-Score

In 1970 Alrete and Kroulik invented a score that should deliver objective information on the physical constitution of patients at the ARU. The sum of five areas that are evaluated respectively from 0 to 2 yields a maximum of 10 [^[[48]](#endnote-49)^].

| Activity | Movement of 4 extremities independently or on request | 2 |
| --- | --- | --- |
|  | Movement of 2 extremities independently or on request | 1 |
|  | No movement of extremities | 0 |
| Respiration | Deep respiration and cough possible | 2 |
|  | Dyspnoea or restricted respiration | 1 |
|  | Apnoea | 0 |
| Blood pressure | RR + 20% of the pre-anaesthetic value | 2 |
|  | RR + 20- 49% of the pre-anaesthetic value | 1 |
|  | RR + 50 % of the pre-anaesthetic value | 0 |
| Consciousness | At full consciousness | 2 |
|  | Can be woken up by calling | 1 |
|  | No response | 0 |
| O_2_ saturation | Oxygen saturation > 92% in room air | 2 |
|  | Requires oxygen to hold oxygen saturation > 90% | 1 |
|  | Oxygen saturation < 90 % in spite of oxygen supply | 0 |

The following tabular document is used for recording the Aldrete scores in the ARU:

Aldrete Score:

| Time (min) | **A** | 05 | 15 | 30 | 45 | 60 | 75 | 90 | 105 | … | **E** |
| --- | --- | --- | --- | --- | --- | --- | --- | --- | --- | --- | --- |
| Activity |  |  |  |  |  |  |  |  |  |  |  |
| Respiration |  |  |  |  |  |  |  |  |  |  |  |
| Blood pressure |  |  |  |  |  |  |  |  |  |  |  |
| Consciousness |  |  |  |  |  |  |  |  |  |  |  |
| O2 saturation |  |  |  |  |  |  |  |  |  |  |  |
| Total points |  |  |  |  |  |  |  |  |  |  |  |

6.7 Cortisol level (hydrocortisone) in the saliva

The glucocorticoid level is subject to daytime and intra-individual variations; however, additional variations can be measured in the case of acute stress conditions [^[[49]](#endnote-50)^, ^[[50]](#endnote-51)^, ^[[51]](#endnote-52)^]. Reliable measurement of cortisol is done by taking saliva sample and it should be done at the beginning and at the end of the ARU care.

6.8 Questionnaire on patient satisfaction

Following assessment of the finale Aldrete score, satisfaction is recorded using the following table in the due course:

Subjective perception (VRS, point values between 0 and 10)

| Time (min) | **A** | 05 | 15 | 30 | 45 | 60 | 75 | 90 | 105 | … | **E** |
| --- | --- | --- | --- | --- | --- | --- | --- | --- | --- | --- | --- |
| Vigilance (drowsy – wide awake) |  |  |  |  |  |  |  |  |  |  |  |
| Well-being |  |  |  |  |  |  |  |  |  |  |  |
| Energy level |  |  |  |  |  |  |  |  |  |  |  |
| Excitement condition |  |  |  |  |  |  |  |  |  |  |  |
| Disorientation |  |  |  |  |  |  |  |  |  |  |  |
| Nausea |  |  |  |  |  |  |  |  |  |  |  |

6.9 Balancing and summary of the ARU stay

Before leaving the ARU, following information is taken from the anaesthesia protocol and made anonymous:

- Drug administrations (drug name, dose, time of administration)
- ARU care period:

For exact documentation content, refer to the annex "Patient file".

7. Safety assessment

The SOM Biofeedback brain coach (CE certificate is present) is used for measurement of the skin conductance. Positive experience with this device can be seen in a previous study (EK 105/20).

CE-certified standard devices (Masimo Radical 7 and Nelcor N600) are used for pulse oximetry. Both signals are read into a notebook using an analogue-digital converter (NI USB-6008, National Instruments) and recorded using a software (National Instruments, Labview, Signal Express). The notebook (Toshiba NB200-11L) is operated over the integrated rechargeable battery (operating time about 8-10 hours).

The saliva samples are taken using the Sarstedt salivettes that were used already in previous studies and do not lead to any stress or risk to the patient.

All other parameters are determined through inquiry and/or clinical, non-invasive examination of the patient or copying from the anaesthesia protocol (NRS, satisfaction, Aldrete score).

Therapeutic consequences (application of analgesics) result exclusively from the inquiry of the patients (NRS), not from the additionally determined readings. All data are recorded completely anonymously.

Therefore, it is to be assumed that the patient will not face any risk.

# 8. Statistics

## 8. 1 Planned statistical methods

First of all, the data of the different study wings (see plan) are reviewed for normal distribution (Kolmogorov-Smirnov test). Any significance between the test and the reference group is assessed using a two-sided T test at the significance level α = 0.05. Besides the comparison of the TC with the KO group, subgroup analyses may be carried out, where appropriate. IBM SPSS Statistics 19 is used for the statistical analyses. Prior to specification of the number of study participants, power analysis is done using nQuery Advisor (STATCON).

## 8.2 Planned number of study participants

Based on a T test of two independent random samples (two-sided), a significance level of α = 0.05 became of and a power of 0.8 is determined.

The effective variable is 0.88 under the assumption of an expected difference of NRS 4 in the TC group and NRS 5 in the KO group and a standard deviation of 25% respectively. Thus, a total random sample of n = 44 (each 22) should be sufficient.

## 8.3 Significance level

The determined significance level amounts to α = 0.05, the power 0.8.

## 8.4 Handling missing, unused and doubtful data

All available data are evaluated independent of any missing data, as far as possible. Doubtful data are excluded from the statistical analysis and are separately discussed.

## 9. Quality inspection and quality assurance

At regular intervals, the original documents are inspected and reviewed jointly by the doctoral candidate and the principal investigators for totality and correctness. For the workflow of the studies, check lists that remain at the patient's place are generated to instruct the involved caretakers and physicians in the ARU about the current status of the study. All original files, documents and Sarstedt salivettes are marked with a patient-specific anonymous ID. All electronically collected data and/or data exports are copied immediately on an external hard disk for security reasons; in addition, regular backups on external storage media are done.

Registration at www.clinicaltrials.gov precedes the clinical study.

## 10. Ethics

The clinical study is preceded by an evaluation by the ethics commission of the University Hospital of Aachen, which is also informed about all changes to the study protocol subject to approval as well as all reportable incidents.

## 11. Handling data and retaining records

Randomisation of a study participant to a group is immediately followed by the allocation of an internal number (ID). After conclusion of the measurements of a patient (leaving the ARU), all records are stored anonymously using his or her ID.

## 12. Funding and insurance

This research project is financed by research funds of the Clinic for Anaesthesiology. The liability insurance of the University Hospital of Aachen was concluded at the Zürich Versicherungs-AG with the insurance policy number 813.380.000.270. Patients are insured if the insurance case is caused due to culpability of the Hospital or one of its staff.

## 13. Referenzen

1. IASP Pain Terminology. International Association for the Study of Pain. http://www.iassp-pain.org/terms-p.html [Accessed January 4, 2011] [↑](#endnote-ref-2)
2. Polomano RC, Dunwoody CJ, Krenzischek DA, Rathmell JP: Perspective on pain management in the 21st century. J PeriAnesth Nurs 2008; 23: 4-14 [↑](#endnote-ref-3)
3. Kehlet H, JensenTS, Woolf CJ. Persistent postsurgical pain: risk factors and prevention. Lancet 2006; 367: 1618-1625 [↑](#endnote-ref-4)
4. Page GG: The immune-supressive effects of pain. Adv Exp Med Biol 2003; 521: 117-125 [↑](#endnote-ref-5)
5. Kehlet H: Surgical Stress: The Role of pain and analgesia. Br J Anaesth 1989; 63: 189-195 [↑](#endnote-ref-6)
6. Vaurio LE, Sands LP, Wang Y, Mullen EA, Leung JM: Postoperative Delirium: The Importance of Pain and Pain Management [↑](#endnote-ref-7)
7. Lynch M: Pain: the fifth vital sign. Comprehensive assessment leads to proper treatment. Advanced Nursing Practice 2001; 9: 28-36 [↑](#endnote-ref-8)
8. Gould TH, Crosby DL, Harmer M, er al: Policy for controlling pain after surgery: effect of sequential changes in management. British Medical Journal 1992; 305: 1187-1193 [↑](#endnote-ref-9)
9. Bosenberg A, Thomas J, Lopez T, Kohinsky E, Larsson LE: Validation of a six graded faces scale for evaluation of postoperative pain in children. Pediatric Anesthesia 2003; 13: 708-713 [↑](#endnote-ref-10)
10. Rodriguez CS, McMillan S, Yarandi H: Pain measurements in older adults with head and neck cancer and communication impairments. Cancer Nursing 2004; 27: 425-433 [↑](#endnote-ref-11)
11. Guingnard B: Monitoring analgesia. Best Practice & Research Clinical Anaesthesiology 2006 20: 161-180 [↑](#endnote-ref-12)
12. Ledowski T, Ang B, Schmarbeck T, Rhodes J: Monitoring of sympathetic tone to assess postoperative pain: skin conductance vs surgical stress index. Anaesth 2009; 64: 727-731 [↑](#endnote-ref-13)
13. Nisbet HIA, Norris W, Brown J: Objective measurement of seadtion, IV. The measurement and interpretation of electrical changes in the skin. Br J Anaest 1967; 39: 798-805 [↑](#endnote-ref-14)
14. Storm H, Shafiei M, Myre K, Raeder J: Palmar skin conductance compared to a developed stress score and to noxious and awakening stimuli on patients in anaesthesia. Acta Anasthesiol Scand 2005; 49: 798-803 [↑](#endnote-ref-15)
15. Storm H: Changes in skin conductance as a tool to monitor nociceptive stimulation and pain. Curr Opin in Anaesthesiol 2008; 21:796-804 [↑](#endnote-ref-16)
16. Storm H, Myre K, Rostrup M, Stockland O, Lien MD, Reader JC: Skin conductance correlates with perioperative stress. Acta Anaesthesiol Scand 2002; 46:887-95 [↑](#endnote-ref-17)
17. Ledowski T, Bromilow J, Paech MJ, Storm H, Hacking R, Schug SA: Skin cinductance monitoring compared with bispectral index to assess emergence from total. i.v. Anaesthesia using propofol and remifentanil. BR J Anaesth 2006; 97: 817-21 [↑](#endnote-ref-18)
18. Ledowski T, Preuss J, Ford A, Paech MJ, McTernan C, Kapila R, Schug SA: New parameters of skin cinductance compared with bispectral index monitoring to assess emergence from total intravenous anaesthesia. Br J Anaest 2007; 99: 547-551 [↑](#endnote-ref-19)
19. Ledowski T, Bromilow J, Paech MJ, Storm H, Hacking R, Schug SA: Skin cinductance monitoring compared with bispectral index to assess emergence from total. i.v. Anaesthesia using propofol and remifentanil. BR J Anaesth 2006; 97: 817-21 [↑](#endnote-ref-20)
20. Ledowski T, Bromilow J, Paech J, er al: Monitoring of skin conductance to assess postoperative pain intensity. BR J Anaest 2006; 97:862-865 [↑](#endnote-ref-21)
21. Ledowski T, Bomilow J, Wu J, Paech MJ, Storm H, Schug SA: The assessmnt of postoperative pain by monitoring conductance: results of a prospective study. Anaest 2007; 62: 989-993 [↑](#endnote-ref-22)
22. Hullett B, Chambers N, Preuss J, Zambudio I, Lange J, Pascoe E, Ledowski T: Monitoring electrical skin conductance. Anaesthesiol 2009; 111: 513-517 [↑](#endnote-ref-23)
23. Gjerstad AC, Storm H, Hagen R, Huiku M, Qvistad E, Raeder J: Comparison of skin conductance with entropy during intubation, tetanic stimulation and emergence from general anaesthesia. Acta Anaesthesiol Scand 2007; 51: 8-15 [↑](#endnote-ref-24)
24. Huiku M, Uutela K, van Gils M, Korhonen I, Kymäläinen M, Meriläinen P, Paloheimo M, Rantanen M, Takala P, Viertiö-Oja H, Yli-Hankala A: Assessment of surgical stress during general anaesthesia. Br J Anaesth 2007; 98( 4): 447-455 [↑](#endnote-ref-25)
25. Ahonen J, Jokela R, Uutela K, Huiku M: Surgical stress index reflects surgical stress in gynaecological laparoscopic day-case surgery. Br J Anaesth 2007; 98 (4): 456-461 [↑](#endnote-ref-26)
26. Struys MMRF, Vanpeteghem C, Huiku M, Uutela K, Blyaert NGK, Mortier EP: Changes in a surgical stress index in response to standardized pain stimuli during propofol-remifentanil infusion. Br J Anaesth 2007; 99 (3): 359-367 [↑](#endnote-ref-27)
27. Kallio H, Lindberg LI, Majander AS, Uutela KH, Niskanen ML, Paloheimo MPJ: Measurement of surgical stress in anaesthetized children. Br J Anaesth 2008; 101 (3): 383-389 [↑](#endnote-ref-28)
28. .  Rohleder N, Wolf JM, Kirschbaum C. Glucocorticoid Sensitivity in Humans-Interindividual Differences and Acute Stress Effects. Stress, 6(3); Sep 2003: 207-22. [↑](#endnote-ref-29)
29. . Hellhammer DH, Wüst S, Kudielka BM. Salivary cortisol as a biomarker in stress research. Psychoneuroendocrinology. 2009 Feb;34(2):163-71. [↑](#endnote-ref-30)
30. . Boudarene M, Legros JJ, Timsit-Berthier M. Study of the stress response: role of anxiety, cortisol and DHEAs. Encephale. 2002 Mar-Apr;28(2):139-46 (nur Abstrakt auf Englisch) [↑](#endnote-ref-31)
31. Merskey H, Bogduk N. Classification of chronic pain, 2nd edn. Seattle: IASP Press, 1994 [↑](#endnote-ref-32)
32. Jensen MP, Turner JA, Romano JM: What is the maximum number of levels needed in pain intesity measurement? Pain. 1994; 58: 387-392 [↑](#endnote-ref-33)
33. Ferrell BA, Ferrell BR, RiveraL: Pain in cognitively impaired nursing home patients. J Pain Symptom Manage. 1995; 10: 591-598 [↑](#endnote-ref-34)
34. Herr KA, Mobily PR: Comparison of selected tools for use with the elderly. Appl Nurs Res. 1993; 6: 39-46 [↑](#endnote-ref-35)
35. Stuppy DJ: The faces pain scale: reliability and validity with mature adults. Appl Nurs Res. 1998; 11: 84-89 [↑](#endnote-ref-36)
36. Manz BD, Mosier R, Nusser- Gerlach MA, Berstrom N, Agrawl S: Pain assessment in the cognitively impaired and unimpaired elderly. Pain Manage Nurs. 2000; 1: 106-115 [↑](#endnote-ref-37)
37. Bini G, Hagbarth KE, Hynninen P, Wallin BG. Thermoregulatory and rhythm generating mechanisms governing the submotor and vasoconstrictor outflow in human cutaneous nerves. J Physiol 1980; 306: 537-552 [↑](#endnote-ref-38)
38. Tranel D, Damasio H: Neuroanatomical correlates of electrodermal skin conductance responses. Psychopathologie 1994; 31: 427-438 [↑](#endnote-ref-39)
39. Edelberg R: Electrical properties of the skin. In: Brown CC, editor. Methods in Psychophysiology. Balti,ore: Williams & Wilkins; 1967. pp 1-59 [↑](#endnote-ref-40)
40. Christine MJ. Electrodermal activity in the 1980's: a review. J R Soc Med 1981; 74: 616-622 [↑](#endnote-ref-41)
41. Gjerstad AC, Storm H, Wallin G: Evaluation of the skin conductance method by using microneurographi. [abstract]. Chicago: ISAP; 2006. [↑](#endnote-ref-42)
42. [↑](#endnote-ref-43)
43. Hagbarth KE, Hallin RG, Hongell A, et al. General characteristics of sympathetic activity in human skin nerves. Acta Physiol Scand 1971; 84: 164-176 [↑](#endnote-ref-44)
44. Wallin BG, Sundlof G, Delius W. The effect of carotid sinus nerve stimulations on muscle and skin nerve sympathetic activity in man. Plugers Arch 1975; 358: 101-110 [↑](#endnote-ref-45)
45. Macfield VG, Wallin BG. The discharge behaviour of single sympathetic outflow in normosensitive human sweat glands. J Auton Nerv Syst 1996; 14: 277-286 [↑](#endnote-ref-46)
46. Ledowski T, Preuss J, Ford A, Paech MJ, McTernan C, Kapila R, Schug SA: New parameters of skin cinductance compared with bispectral index monitoring to assess emergence from total intravenous anaesthesia. Br J Anaest 2007; 99: 547-551 [↑](#endnote-ref-47)
47. Harrison D, Boyce S, Loughnan P, Dargaville P, Storm H, Johnston L: Skin conductance as a measure of pain and stress in hospitalized infants. Early Hum Dev 2006; 82: 603-608 [↑](#endnote-ref-48)
48. Aldrete JA, Kroulik D: A Postanaesthetic Recovery Score. Anaesthesia and Analgesia 1970; 49: 924-934 [↑](#endnote-ref-49)
49. .  Rohleder N, Wolf JM, Kirschbaum C. Glucocorticoid Sensitivity in Humans-Interindividual Differences and Acute Stress Effects. Stress, 6(3); Sep 2003: 207-22. [↑](#endnote-ref-50)
50. . Hellhammer DH, Wüst S, Kudielka BM. Salivary cortisol as a biomarker in stress research. Psychoneuroendocrinology. 2009 Feb;34(2):163-71. [↑](#endnote-ref-51)
51. . Boudarene M, Legros JJ, Timsit-Berthier M. Study of the stress response: role of anxiety, cortisol and DHEAs. Encephale. 2002 Mar-Apr;28(2):139-46 (Abstrakt)

    ## 14. Signatures

    | Dr Michael Czaplik  Principal investigator | Fatima Kezze  Investigator |
    | --- | --- |
    | Dr Julia Kaliciak  Investigator | Christa Hübner  Medical doctoral candidate |
    | Prof Dr Rolf Rossaint  Director of Department for Anaesthesiology |  |

    [↑](#endnote-ref-52)
